# Impact of coronary artery disease on contractile function and ventricular‐arterial coupling during exercise: Simultaneous assessment of left‐ventricular pressure–volume and coronary pressure and flow during cardiac catheterization

**DOI:** 10.14814/phy2.14768

**Published:** 2021-05-27

**Authors:** Tiffany Patterson, Simone Rivolo, Daniel Burkhoff, Jan Schreuder, Natalia Briceno, Rupert Williams, Satpal Arri, Kaleab N. Asrress, Christopher Allen, Jubin Joseph, Hannah Z. R. McConkey, Howard Ellis, Antonis Pavlidis, Brian Clapp, Divaka Perera, Jack Lee, Michael S. Marber, Simon R. Redwood

**Affiliations:** ^1^ Cardiovascular Division King's College London St. Thomas’ Hospital London UK; ^2^ Department of Imaging Science King's College London St. Thomas’ Hospital London UK; ^3^ Cardiac Research Foundation New York NY USA; ^4^ CD Leycom Erasmus University Rotterdam Rotterdam The Netherlands; ^5^ Cardiothoracic Department St. Thomas’ Hospital Guy’s and St. Thomas’ NHS Foundation Trust London UK

**Keywords:** exercise physiology, ischemic heart disease, pressure–volume loop, ventricular‐arterial coupling

## Abstract

Coronary artery disease (CAD) can adversely affect left ventricular (LV) performance during exercise by impairment of contractile function in the presence of increasing afterload. By performing invasive measures of LV pressure–volume and coronary pressure and flow during exercise, we sought to accurately measure this with comparison to the control group. Sixteen patients, with CCS class >II angina and CAD underwent invasive simultaneous measurement of left ventricular pressure–volume and coronary pressure and flow velocity during cardiac catheterization. Measurements performed at rest were compared with peak exercise using bicycle ergometry. The LV contractile function was measured invasively using the end‐systolic pressure–volume relationship, a load independent marker of contractile function (Ees). Vascular afterload forces were derived from the ratio of LV end‐systolic pressure to stroke volume to generate arterial elastance (Ea). These were combined to assess cardiovascular performance (ventricular‐arterial [VA] coupling ratio [Ea/Ees]). Eleven patients demonstrated flow‐limiting (FL) CAD (hyperemic Pd/Pa <0.80; ST‐segment depression on exercise); five patients without flow‐limiting (NFL) CAD served as the control group. Exercise in the presence of FL CAD was associated impairment of Ees, increased Ea, and deterioration of VA coupling. In the control cohort, exercise was associated with increased Ees and improved VA coupling. The backward compression wave energy directly correlated with the magnitude contraction as measured by dP/dTmax (*r* = 0.88, *p* = 0.004). This study demonstrates that in the presence of flow‐limiting CAD, exercise to maximal effort can lead to impairment of LV contractile function and a deterioration in VA coupling compared to a control cohort.

## Graphical abstract

This invasive catheter laboratory exercise study in patients with ischemic heart disease demonstrated impairment of LV contractile function and deterioration in in VA coupling on maximal exercise in the presence of flow‐limiting coronary artery disease.

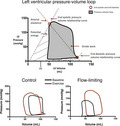

## 1 INTRODUCTION

In the presence of flow‐limiting (FL) coronary artery disease (CAD), adaptations in coronary microvascular resistance are exhausted, thus limiting the necessary increase in coronary blood flow during exercise Bache & Cobb, 1977; Duncker & Bache, 2008). This supply‐demand mismatch can result in ischemia with an adverse effect on left ventricular performance through impairment of contractile function in the presence of increasing forces of afterload Collins, (1990); Carroll et al., 1983). The impact of exercise on left ventricular performance in the presence of FL coronary disease is poorly understood (Asrress et al., 2017; Lockie et al., 2012). Previous limitations include the use of surrogate measures of left ventricular mechanics and an inability to convey the impact on cardiovascular performance and the ventricular–arterial interaction (Grossman et al., 1980; Paulus et al., 1985).

**FIGURE 1:**
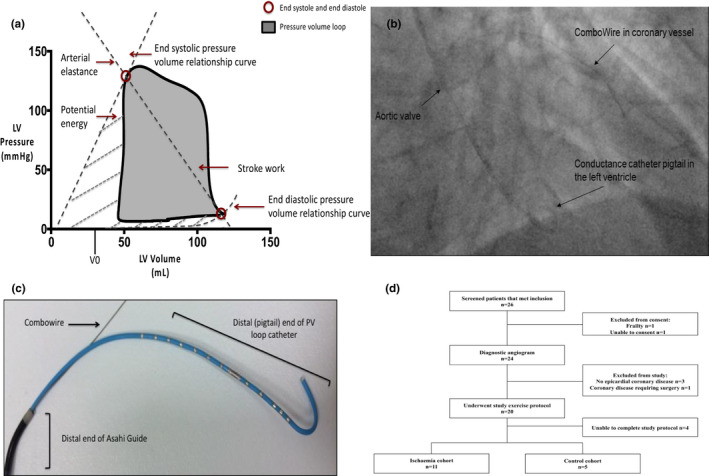
Data analysis using the PV loop, in‐vivo acquisition, and patient flow diagram. (a) The pressure–volume (PV) loop and derived measurements. The pressure–volume loop describes a single cardiac cycle as left ventricular pressure as a function of volume and facilitates an understanding of the cardiac hemodynamics. The end‐systolic pressure–volume relationship (ESPVR) line slope (Ees) represents the load‐independent contractile function of the heart. The end‐diastolic pressure–volume relationship (EDPVR) line slope (EDPVR) represents the load‐independent diastolic properties of the heart. The area of the pressure–volume loop represents stroke work (SW), and the combination of SW and potential energy (boundary from ESPVR) represents the total pressure–volume area (PVA), Arterial elastance is the ratio of end‐systolic pressure to stroke volume (Ea). (b) In‐vivo fluoroscopic image of real‐time intracoronary and intraventricular data acquisition. Fluoroscopy was used to confirm (PA view) correct positioning of Combowire in the coronary artery (left anterior descending) and conductance catheter in the apex of the left ventricle. (c) Ex vivo image of the Asahi sheathless guide with CC and Combowire. (d) Patient flow diagram

Invasive left ventricular pressure–volume (PV) analysis is the gold standard method for assessing the left ventricular contractile function, the end‐systolic pressure–volume relationship has been shown to provide the most accurate load‐independent measure of LV function (Figure 1a). PV analysis also enables accurate determination of LV afterload known as arterial elastance (Ea). Ea is derived from the ratio of LV end‐systolic pressure to stroke volume Baan et al., (1984). Left ventricular performance can then be mathematically quantified by measuring the ratio between afterload (Ea) and LV contractile function (Ees) derived from the PV loop. This interaction is termed ventricular‐arterial coupling (Ea/Ees), and the cardiovascular performance is optimal when the Ea/Ees ratio is less than or equal to 1.0 (Steendijk et al., (2006); Asanoi et al., 1989). Advances in technology have enabled these measurements to be accurately performed in humans; these have been previously validated and applied in the study of cardiac disease states (Burkhoff et al., 1985; Gaemperli et al., 2013; Kapur et al., 2013; Velde et al., 1992).

Coronary wave intensity analysis (WIA) derived from coronary pressure and flow velocity signals can help further identify the transmission of aortic and cardiac contractile forces on coronary blood flow by separating the transmission of energy into forward and backward waveforms. The backward compression wave (BCW) is generated by an increase in myocardial contractile force occurring early in systole during isovolumic contraction, this opposes forward coronary flow and is therefore responsible for early systolic deceleration in coronary blood flow velocity.

The impact of exercise on cardiovascular performance (Ea/Ees ratio) in the presence of FL CAD has not been examined in humans. This study compared the effects of exercise on coronary blood flow velocity and LV contractile function and therefore cardiovascular performance in patients with and without FL CAD by performing invasive measures of LV pressure–volume and coronary pressure and flow during dynamic exercise in patients with normal resting left ventricular function. Furthermore, we examine the interaction between increasing LV contractile force during exercise (dP/dt max) and coronary blood flow deceleration as measured by the BCW. We hypothesized that exercise in the presence of FL CAD would lead to a deterioration in cardiovascular performance compared to a cohort without flow‐limiting CAD.

## 2 METHODS

### 2.1 Study population

This prospective observational study was conducted at St Thomas’ Hospital, London, United Kingdom. Participants were considered eligible for the study if they had symptomatic stable angina CCS Class II‐III, with LV ejection fraction>50% followed by identification of >50% stenoses in ≥1 epicardial coronary artery. Participants were excluded from the study if they had previous coronary artery bypass graft surgery, severe aortic stenosis, severe multi‐vessel coronary disease requiring coronary artery bypass graft surgery, and chronic total occlusions. Oral vasoactive preparations were stopped 48 h prior to the procedure. All patients underwent written, informed consent prior to partaking in the study. This study was deemed safe and received approval from the National Research Ethics Committee (08/H0802/136).

### 2.2 Experimental protocol and instrumentation

Left heart catheterization was performed via the right radial artery using a standard 5Fr arterial sheath. Weight adjusted heparin was administered (70 IU/kg) intra‐arterially. Right and left coronary angiograms were then performed using standard diagnostic catheters. Following angiographic identification of coronary stenosis, a 7.5Fr sheathless guide (Asahi, Vascular Perspectives) was introduced into the aortic root and engaged in the coronary ostium. A dual‐sensor pressure–velocity 0.014” coronary guide wire (Combowire, Volcano Corp) was advanced to the coronary artery via the guide catheter. Following normalization of the pressure signal (Pd/Pa=1.00), the Combowire was advanced distal to the stenosis in the target coronary artery to enable the invasive measurement of distal coronary pressure (Pd) and coronary blood flow velocity (U) (Lockie et al., 2012; Siebes et al., 2004). Left ventricular pressure and volume measurements were performed with a 4Fr conductance catheter (CC) system (CD Leycom, The Netherlands). This flexible pigtail catheter has a solid‐state pressure sensor and electrodes situated at regular intervals, and time‐varying conductance was used to calculate continuous left ventricular volumes. The guide catheter was disengaged from the coronary ostium, and the CC was connected to a signal processor (Inca, CD Leycom) and delivered to the ventricular cavity across the aortic valve via the same guide catheter. Correct positioning was confirmed by fluoroscopy (Figure 1b) and conductance signals. Dynamic exercise was performed using a supine bicycle ergometer (Ergosana, Germany) that adapted for use on the catheter laboratory table. The protocol was a standardized program, starting at 30 W and increasing incrementally by 20 W per minute, performed for 5 min. Exercise was terminated if any of the following occurred: (1) ST segment depression >3 mm, (2) severe chest pain, (3) physical exhaustion, and (4) sustained arrhythmia (Asrress et al., 2017; Lockie et al., 2012).

Measurements were performed during steady‐state conditions, avoiding excessive arrhythmia from premature beats. Recorded variables were averaged from five cardiac cycles. Following the experimental protocol, patients underwent physiological assessment of the coronary stenosis retrospectively with intravenous adenosine and underwent angioplasty where necessary (De Bruyne et al., 2012).

### 2.3 Data acquisition and analysis

**FIGURE 2:**
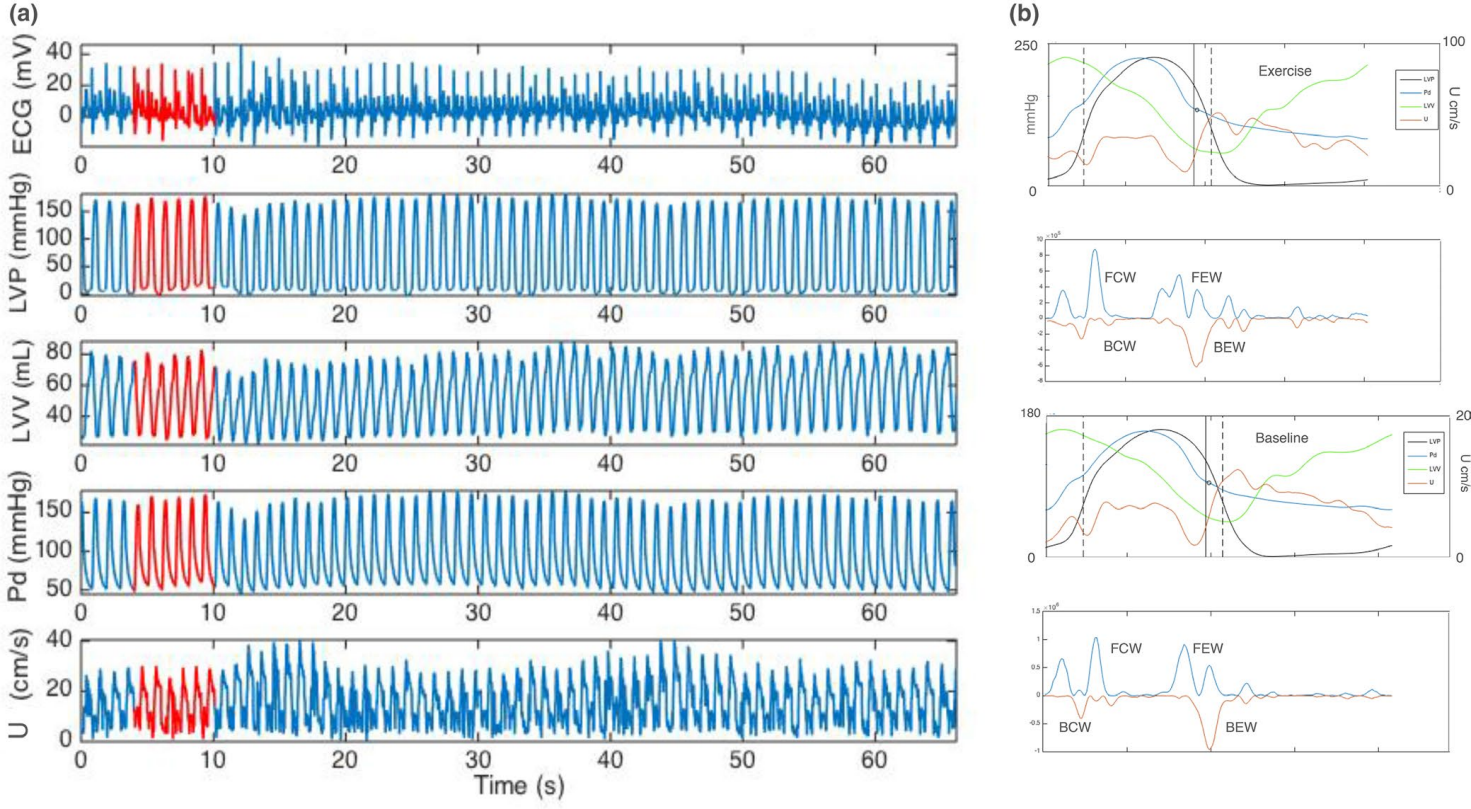
Simultaneous coronary and LV data acquisition. (a) Representative example of simultaneous coronary and LV hemodynamic measurements at baseline as a function of time with 6 beat ensemble average (red). From top to bottom, the panels display continuous intra‐cardiac ECG recording, left ventricular pressure (LVP), left ventricular volume (LVV), distal coronary pressure (Pd), and mean coronary flow velocity (U). (b) demonstrates simultaneous coronary and LV hemodynamic measurements at the baseline and during exercise averaged over one cardiac cycle and coronary wave energies at the baseline and during dynamic exercise

Due to the effects of fatigue during the exercise period, peak exercise was measured at the point of maximal power output (joules per second) derived from the heart rate and the PV loop area. Continuous ECG recordings, left ventricular hemodynamic tracings, and coronary pressure and flow velocity signals were recorded throughout the intervention. A visual example of simultaneous LV and coronary hemodynamic tracings over time is provided in Figure 2a, and simultaneous LV and coronary hemodynamics are also presented over one cardiac cycle in Figure 2b.

Coronary pressure and flow velocity signals (Combomap system, Volcano Corp) and CC measurements were acquired in real‐time (Conduct NT, version 3.18.1 CD Leycom). The mean coronary blood flow velocity (U) and distal coronary pressure (Pd) were determined from the Doppler signal and high‐fidelity pressure signal, respectively, distal to the coronary stenosis. The coronary flow reserve was calculated as the ratio of coronary flow velocity during hyperemia/baseline coronary flow velocity.

Figure 1a demonstrates the pressure–volume (PV) loop and corresponding points on the loop that data were acquired. End‐diastole was measured at the peak of the R wave on the ECG, and end‐systole was measured at the point of maximum pressure/volume in the cardiac cycle for consistency. The end‐diastolic volume (EDV) and end‐systolic volume (ESV) were measured from the PV loop. The stroke volume was calculated as the difference in these volumes. The end‐systolic pressure (ESP) and end‐diastolic pressure (EDP) were also measured directly from the PV loop. These baseline measurements were then used to calculate various parameters related to LV performance Gaemperli et al., 2013. The end‐systolic pressure–volume relationship curve has previously been shown to be independent of volume loading, within a physiological range, and is the most accurate measure of LV contractile function. This was calculated as the ratio of ESP to ESV, referred to as Ees _(single point, SP)_. The volume‐axis intercept (Vo) of the ESPVR was assumed to be 0 (IVC occlusion was not performed in patients, and the single beat method has not been validated in patients during exercise and produced measurements that were outside of the physiological range) Klotz et al., 2006; Chen et al., 2001). The slope of the end‐diastolic pressure–volume relationship was used to describe the LV diastolic function and was calculated using the equation EDP =An.EDV (Bn) with An =28.2 mm Hg and Bn =2.79; the values are expressed as the alpha and beta coefficients of the equation (Klotz et al., (2006)). The LV stroke work that is the external work produced by the ventricle was calculated from the area of the PV loop (Figure 1a) (Gaemperli et al., 2013; Steendijk et al., 2006). Effective Ea is the ratio of SV to end‐systolic pressure and combines the steady and pulsatile components of left ventricular afterload. When combined with Ees_(SP)_, this can be used to accurately quantify ventricular‐arterial coupling, which was reported as the Ea:Ees_(SP)_ ratio. The pressure–volume area (PVA) represents the total cardiac work and was estimated as the sum of stroke work (SW) and elastic potential energy (Figure 1a) and has previously been validated as a surrogate for myocardial oxygen consumption. The LV mechanical efficiency was determined by the ratio of effective stroke work to overall cardiac work (SW/PVA) expressed as a percentage.

For the purposes of determining relationships between coronary wave energies and indices of cardiac contraction, wave intensity analysis (WIA) was performed using time derivatives obtained after smoothing the raw signals using an adaptive Savitzky–Golay filter to improve WIA robustness, which has previously been described Rivolo, Asrress, et al., 2014; Rivolo et al., 2014). The net wave intensity (dI) was performed and normalized to the sampling rate, and separation into forward and backward components was performed using the single‐point technique (Davies et al., 2006; Parker, 2009). The baseline pulse wave velocity was implemented for WIA determination during exercise to account for limitations in the single‐point technique during hyperemia (Rolandi et al., 2014). We examined the association between LV contraction (dP/dTmax) and the backward compression wave (FCW), which has previously been described as associated with the early systolic deceleration of coronary blood flow during isovolumic contraction, in both the control group and patients with FL CAD.

### 2.4 Statistical analysis

Cardiovascular performance is optimal when the Ea/Ees ratio is less than or equal to 1.0. Thus, we determined that a sample size of 20 patients would be necessary to demonstrate a statistically significant mean difference of Ea/Ees of 0.5 (two‐sided alpha 0.05, 80% power) at peak exercise between the two cohorts. Quantitative data are expressed as mean (SD) and median (IQR); categorical variables are described as proportions and percentages. Data were assessed for normality of (Gaussian) distribution both graphically and using the Shapiro–Wilk test. The statistical comparison of serial hemodynamic measurements (quantitative data) of normal distribution within subjects was performed using paired *t*‐tests. Statistical comparison between subjects was performed using a repeated measures one‐way ANOVA, and adjustment for multiple comparisons was performed using the Bonferroni correction to explain significant differences. Categorical data were compared using the Pearson's chi‐squared test. A value of *p* < 0.05 was considered statistically significant for all tests. Statistical analysis was performed using SPSS v24.0 (IBM).

## 3 RESULTS

Between December 2013 and May 2016, 26 patients were consented into the study and underwent diagnostic coronary angiography. Of these patients, reasons for exclusion were as follows: no epicardial coronary disease, three‐vessel coronary disease requiring coronary artery bypass graft surgery, and inability to complete the study protocol due to technical reasons. A total of 16 patients completed the study protocol (Figure 1d). Of the 16, 11 patients demonstrated FL coronary artery lesions (median fractional flow reserve (FFR) 0.62; IQR 0.6 to 0.75) with ST‐segment depression on exercise compared to rest (−50±31mv vs. −23±15mv; *p* < 0.05). Of the 16 patients, 5 did not have flow‐limiting (NFL) coronary lesions (median FFR 0.92 [IQR 0.87 to 0.97]) and did not exhibit significant ST‐segment depression on exercise compared to rest (−9±8mv vs. −7±9mv); these served as the control group. We did not demonstrate a difference in coronary flow velocity between the cohorts. CFR was numerically less in the presence of FL CAD compared to control, but this did not reach significance (1.4 ± 0.7 vs 1.6 ± 0.8). There were no other significant differences in baseline characteristics between the groups (Table 1).

**TABLE 1:** Baseline characteristics of all the study participants (*n* = 31 in total) and divided per study arm: (1) Exercise with non‐flow‐limiting (NFL) coronary artery disease and (2) exercise with flow‐limiting (FL) coronary artery disease and nitroglycerin

	Control group (5)	Flow‐limiting coronary disease (11)	*p*
Male sex	5 (100)	8 (72.7)	0.439
Age, years	65 ± 10.1	67.7 ± 11.1	0.908
Height, cm	168.2 ± 4.3	167.8 ± 9.9	0.842
BMI kg/m^2^	31.5 ± 5.2	27.9 ± 3.8	0.212
Previous PCI	4 (80)	4 (36.4)	0.228
Previous MI	1 (20)	2 (18.2)	0.649
LVEF, %	58.2 ± 6.6	56.1 ± 8.8	0.858
Diabetes mellitus	3 (60)	6 (54.5)	0.431
Hypertension	4 (80)	9 (81.8)	0.649
Hypercholesterolemia	5 (100)	9 (81.8)	0.602
Family History	4 (80)	6 (54.5)	0.431
Smoking history	4 (80)	7 (63.6)	0.597
Current Medications			
Βeta‐blocker	4 (80)	6 (54.5)	0.936
Long‐acting nitrate	1 (20)	3 (27.3)	0.644
Statin	5 (100)	8 (72.7)	0.439
ACEi/AIIRB	2 (40)	8 (72.7)	0.435
Ca channel antagonist	1 (20)	5 (45.5)	0.488
Nicorandil	1 (20)	1 (9.1)	0.681
Aspirin	4 (80)	10 (90.9)	0.681
Clopidogrel	3 (60)	8 (72.7)	0.187
Diseased vessels	2 (1 to 2)	2 (1 to 2)	0.480
Hyperemic Pd/Pa (FFR)	0.92 (0.87 to 0.97)	0.62 (0.6 to 0.75)	<0.001*
LAD/Cx/RCA	3/1/2	5/1/4	—
Duration (mins)	97 (73.5 to 114)	97 (82 to 112)	0.075

Normally distributed continuous data are displayed as mean ±SD, continuous data that are not normally distributed are presented as median (IQR), and categorical data are presented as n (%), where n is the number of patients in that study group with a certain characteristic.

Abbreviations: ACEi, angiotensin converting enzyme inhibitor; AIIRB, angiotensin II receptor blocker; BMI, body mass index; Cx, circumflex artery; LAD, left anterior descending artery; LVEF, left ventricular ejection fraction; MI, myocardial infarction; PCI, percutaneous coronary intervention; Pd/Pa, mean distal coronary pressure/man aortic pressure; RCA, right coronary artery.

* Indicates the *p* value <0.05

### 3.1 Control group

**FIGURE 3:**
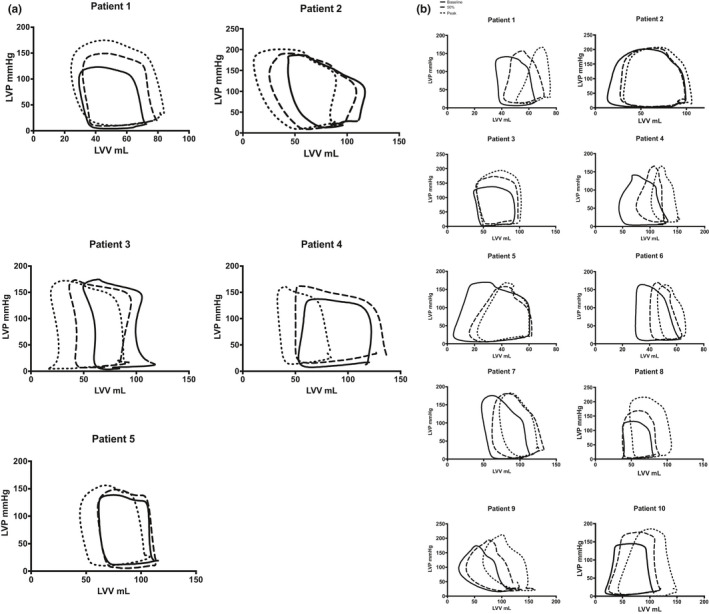
Pressure–volume loops at rest and on exercise. (a) Pressure–volume diagrams in patients with non‐flow‐limiting coronary artery disease at rest (solid line) at 50% peak exercise (broken line) and peak exercise (dotted line). All patients demonstrated an improved contractile function and mechanical efficiency (SW:PVA ratio), with a leftward shift of the PV loop and a leftward upward shift of the end‐systolic pressure–volume relationship. (b) Pressure–volume diagrams in patients with flow‐limiting coronary artery disease at rest (solid line) at 50% peak exercise (broken line) and peak exercise (representing ischemia (dotted line). Ischemia was associated with a rightward shift of the ESPVR and a rightward, upward shift of the end‐diastolic pressure–volume relationship (EDPVR). A rightward shift of the PV loop is consistent with a decrease in LV efficiency; a reduction in the stroke work (PV loop area) to pressure–volume area (stroke work +potential energy) ratio

In the control group cohort, there was a significant increase in heart rate (101 ± 5 vs. 85 ± 9 bpm; *p* < 0.05) and left ventricular pressure (EDP 32.9 ± 6.6 vs. 19.8 ± 6.3 mmHg; *p* < 0.05) on exercise compared to rest. There was a significant increase in ejection fraction (73 ± 14 vs. 57 ± 6%; *p* < 0.05) and contractile function, as evidenced by a significant increase in dP/dt max (1911 ± 124 vs. 1415 ± 173; *p* < 0.05) and Ees_(SP)_ (5.3 ± 3.0 vs. 2.6 ± 1.2; *p* < 0.05) on exercise compared to rest. There was no change in Ea; however, there was a significant improvement in the ventricular‐arterial coupling ratio (Ea:Ees ratio 0.5 ± 0.2 vs. 1.0 ± 0.3; *p* < 0.05) and an increase in cardiac efficiency (SW:PVA 76.2 ± 8.8 vs. 61.0 ± 7.5%; *p* < 0.05) on exercise compared to rest. There were no significant changes on load‐independent measures of diastolic function on exercise compared to rest (EDPVR). Pressure–volume diagrams for individual patients in the control cohort at rest and on exercise are provided in Figure 3a. All measured and derived LV and coronary hemodynamic indices and pressure–volume derived indices are provided in Table 2 for both cohorts.

**TABLE 2:** All measured and derived LV and coronary hemodynamic indices and pressure–volume derived indices in participants in both cohorts: control group and flow‐limiting coronary artery disease

	Control group (5)		Flow‐limiting coronary disease (11)	
	Baseline	Peak	Baseline	Peak
LV hemodynamic indices				
HR bpm	85 ± 9	101 ± 5*	77 ± 14	104 ± 20*
ST deviation (mV)	−7 ± 9	−9 ± 8	−23 ± 15	−50 ± 31*
EDV mL	106.3 ± 19.0	86.7 ± 8.5	91.4 ± 25	106.9 ± 34.3*
ESV mL	49.1 ± 13.5	27 ± 13.6*	38.3 ± 13.2	61.2 ± 23.4*
EF %	57 ± 6	73 ± 14*	69 ± 16	52 ± 16*
SV	63.0 ± 13.3	67.6 ± 12.9	67.1 ± 26.4	58.9 ± 30.1*
EDP mmHg	19.8 ± 6.3	32.9 ± 6.6*	21.2 ± 14.5	26.9 ± 8.8
ESP mmHg	142.2 ± 28.7	152.2 ± 19.0	141.3 ± 16.9	158.7 ± 16.6
dP/dt_max_	1415 ± 173	1911 ± 124*	1464 ± 283	1791 ± 601
dP/dt_min_	−1631 ± 307	−1883 ± 232	−1523 ± 278	−1926 ± 399*
Pressure–volume derived indices				
E_es SP_	2.6 ± 1.2	5.3 ± 3.0*	3.1 ± 1.3	2.8 ± 0.9
E_a_	2.3 ± 0.3	2.3 ± 0.3	2.4 ± 1.1	3.4 ± 1.3*
E_a_/E_es SP_	1.0 ± 0.3	0.5 ± 0.2*	0.9 ± 0.4	1.3 ± 0.8*
SW	6605 ± 1994	8351 ± 2248	7169 ± 17	7707 ± 4489
PVA	10872 ± 2972	10811 ± 1680	10071 ± 5048	11814 ± 6682
SW:PVA	61.0 ± 7.5	76.2 ± 8.8*	63.8 ± 11.4	56.4 ± 14.5*
β EDPVR_(SB)_	6.1 ± 0.1	4.7 ± 2.5	5.4 ± 1.2	5.9 ± 2.5
Tau	29.6 ± 3.8	27.7 ± 3.6	32.8 ± 6.1	29.0 ± 6.2*
Coronary hemodynamic indices				
U cm/s	11.4 ± 3.2	18.4 ± 6.6	12.1 ± 6.3	20.1 ± 12.1
Pd mmHg	108.1 ± 10.3	120.0 ± 14.7	94.5 ± 11.9	107.8 ± 24.1
CFR	—	1.6 ± 0.8	—	1.4 ± 0.7
BCW	−5246 ± 3531	−7611 ± 3463	−5421 ± 3086	−10571 ± 5353

Peak exercise was calculated as the maximum power output (J/second) derived from the pressure–volume loop area and heart rate. Data are displayed as mean ±SD.

Abbreviations: BCW, backward compression wave; CFR, coronary flow reserve; Ea, arterial elastance; EDP, end‐diastolic pressure; EDV, end‐diastolic volume; E_es_, end‐systolic elastance; EF, ejection fraction, ESP, end‐systolic pressure; ESV, end‐systolic volume; HR, heart rate; Pd, mean distal coronary pressure; PVA, pressure–volume area; SV, stroke volume; SW, stroke work; U, mean coronary blood flow velocity; β EDPVR_(SB)_, single beat estimate of the beta coefficient of the end‐diastolic pressure–volume relationship curve.

* Indicates the *p* value <0.05

### 3.2 Flow‐limiting coronary disease

In the cohort of patients with physiologically significant FL CAD, there was a significant increase in heart rate (104 ± 20 vs. 77 ± 14 bpm; *p* < 0.05) and LV volumes (EDV 106.9 ± 34.3 vs. 91.4 ± 25 ml; *p* < 0.05 and ESV 61.2 ± 23.4 vs. 38.3 ± 13.2 ml; *p* < 0.05) on exercise compared to rest. There was a significant decrease in ejection fraction (52 ± 16 vs. 69 ± 16; *p* < 0.05) on exercise compared to rest, but there was no change in load‐independent measures of contractile function. There was an increase in Ea (3.4 ± 1.3 vs. 2.4 ± 1.1; *p* < 0.05) with a decline in the ventricular‐arterial coupling ratio (Ea:Ees 1.3 ± 0.8 vs. 0.9 ± 0.4; *p* < 0.05) and cardiac efficiency (SW:PVA 56.4 ± 14.5 vs. 63.8 ± 11.4%; *p* < 0.05) on exercise compared to rest. There were no significant changes on load‐independent measures of diastolic function on exercise compared to rest (EDPVR). Pressure–volume diagrams for individual patients in the ischemia cohort at rest and on exercise are provided in Figure 3b.

### 3.3 Coronary hemodynamics and wave intensity analysis

In the control group cohort, there was a numerical increase in coronary blood flow velocity and distal coronary pressure on exercise compared to baseline, but this did not reach significance. Similarly, there was a numerical increase in the BCW energies on exercise compared to baseline (−7611 ± 3463 vs. −5246 ± 3531; *p* = 0.078); however, this did not reach significance.

In the cohort of patients with FL CAD, there was a numerical increase in coronary blood flow velocity and distal coronary pressure on exercise compared to baseline, but this did not reach significance. CFR was numerically less in the presence of FL CAD compared to control, but this did not reach significance (1.4 ± 0.7 vs. 1.6 ± 0.8). Similarly, there was a numerical increase in the BCW energies on exercise compared to baseline (−10571 ± 5353 vs. −5421 ± 3086; *p* = 0.053); however, this did not reach significance. However, on analysis of both the control group and FL CAD cohorts, there was a significant increase in BCW wave energy on exercise compared to baseline (−9584 ± 4861 vs. −5096 ± 3078; *p* = 0.019).

**FIGURE 4:**
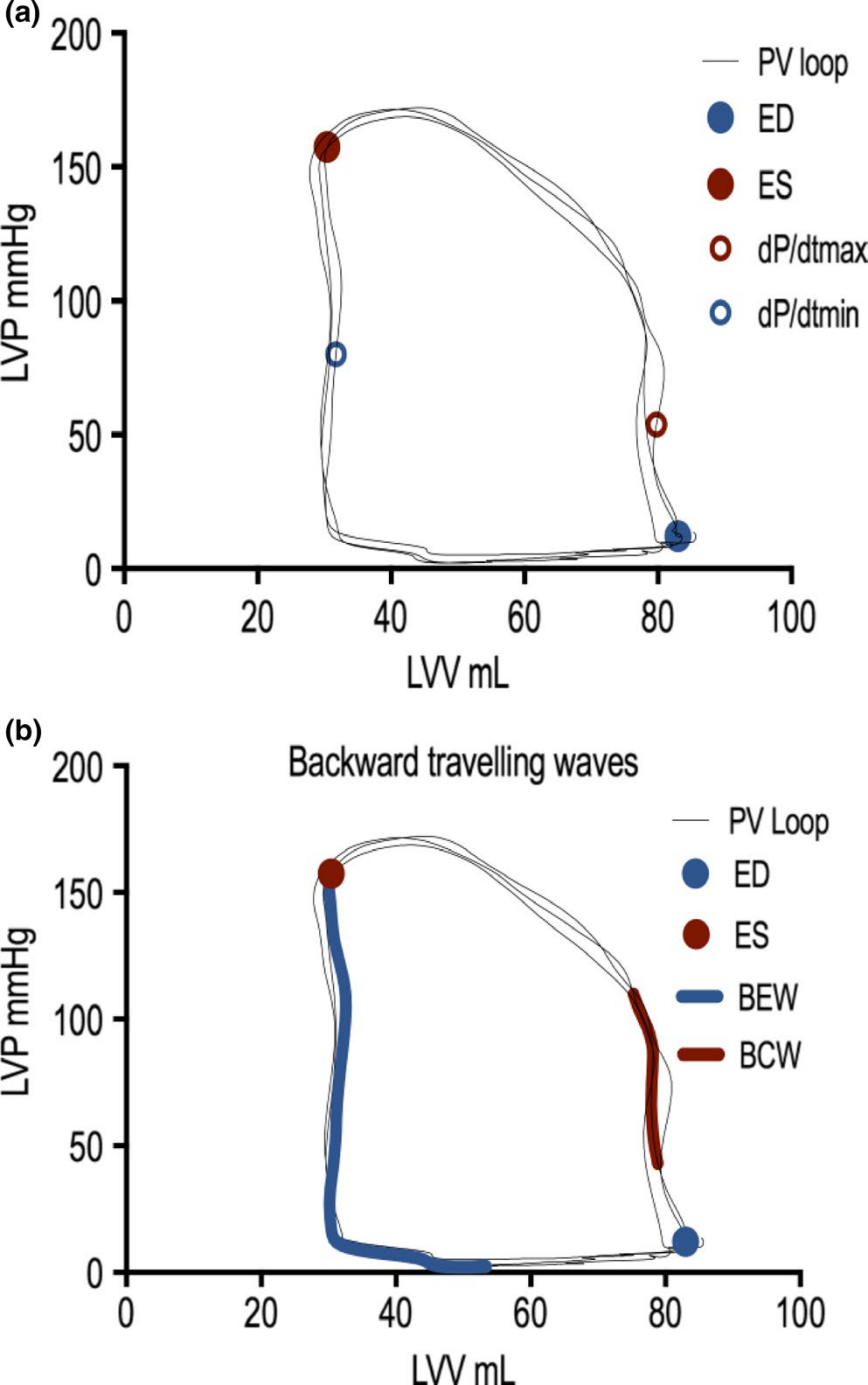
Coronary wave energies superimposed on the pressure–volume (PV) loop. (a) The cardiac cycle depicted as LV pressure (LVP) as a function of volume (LVV), the end‐diastole (ED), end‐systole (ES), and dP/dT max and dP/dTmin (maximal rate of pressure increase and pressure decline respectively) depicted on the PV loop. (b) The backward compression wave (BCW) originates during isovolumic contraction and terminates immediately after aortic valve opening (when the LV pressure exceeds the aortic diastolic pressure). The backward expansion wave (BEW) originates on the aortic valve closure, at end‐systole, and terminates at a minimum LV pressure

**FIGURE 5:**
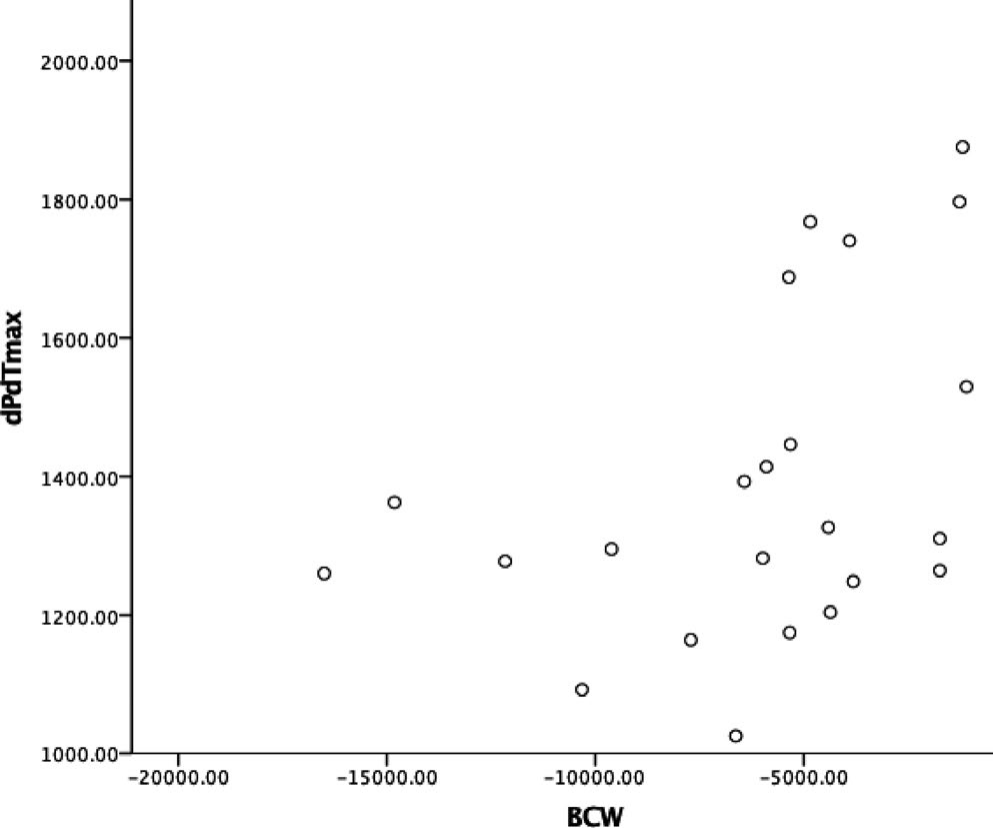
Scatter plot. This demonstrates the correlation between backward compression wave energy and dP/dTmax at rest in the cohort of patients with flow‐limiting coronary artery disease

Simultaneous analysis of the cardiac cycle and coronary flow enabled depiction of the timings of the cardiac wave energies relative to the cardiac cycle and also dP/dt max and dP/dt min (Figure 4a and b). This novel depiction of wave energies relative to the pressure–volume loop is hypothesis generating with regard to the temporal alignment of dP/dt max to BCW. We therefore sought to examine the relationship between the BCW with LV contraction as measured by dP/dtmax. On simultaneous analysis of PV loop and coronary wave energies, LV contraction as measured by dP/dT max correlated with backward compression wave energy in the coronary artery at rest (*r* = 0.88, *p* = 0.004). The correlation was maintained when both cohorts were analyzed together (control group and FL CAD: *r* = 0.75, *p* = 0.005) (Figure 5). This correlation was not demonstrated on exercise.

## 4 DISCUSSION

This mechanistic study demonstrated the effect of exercise on left ventricular contractile function and cardiovascular performance in patients with and without FL coronary disease. The two main observations were as follows: (1) In the absence of FL coronary disease, exercise improved left ventricular contractile function and ventricular‐arterial coupling, thus increasing cardiac efficiency; (2) FL coronary disease was associated with impairment of contractile function and deterioration in ventricular‐arterial coupling, thus decreasing cardiac efficiency. The findings of our study support and thus confirm those of earlier preclinical studies of exercise in the presence of both FL and non‐flow‐limiting coronary diseases with the addition of ventricular‐arterial coupling Nozawa et al., (1994); Little et al., 1989; Steendijk et al., 1998). A greater understanding of the mechanisms of ischemia facilitates development of targeted therapies in this cohort.

Ventricular‐arterial coupling is an important measure of cardiac efficiency, and in the cohort of patients with FL CAD with normal resting LV function, we demonstrated a mismatch in the Ea:Ees ratio on exercise and also a reduction in the SW:PVA. This would suggest a decrease in cardiac efficiency and overall decrease in cardiovascular performance. Furthermore, left ventricular volumes increased with increasing heart rate and exercise in the cohort of patients with FL CAD. This could be hypothesized to be a compensatory mechanism in the absence of an increase in contractile function in order to maintain the cardiac output. This was in contrast to the control group that demonstrated increased contractile function and a decrease in end‐systolic volume on exercise compared to baseline. Furthermore, the control group demonstrated improved ventricular‐arterial coupling and SW:PVA ratio and therefore an increase in cardiac efficiency. Elevated end‐diastolic pressures were observed on exercise in both the control group and in patients with ischemia. In the control group, this would have likely been attributable to impaired diastolic function (associated with comorbidities and increasing age) but also the leg raise required to perform supine bicycle exercise.

Interestingly, we did not observe statistically significant differences in coronary blood flow velocity despite numerical increases in either cohort on exercise compared to rest, and we hypothesize that this was due to the small numbers in each cohort. The observed changes in LV function during ischemia were pronounced and secondary to supply‐demand mismatch and therefore presumed secondary to FL coronary disease. Although coronary flow velocity at peak exercise did not appear different between the cohorts, a FFR of <0.8 and lower CFR value in the ischemic group were assumed to represent FL coronary disease. CFR was numerically less in the cohort of patients that demonstrated ischemic changes on exercise. However, this difference did not reach significance and was further cofounded by the likely co‐existence of microvascular disease in patients with diabetes and hypertension, which also reduces CFR. Furthermore, a mean CFR value of <2 in the control group may explain symptoms of chest discomfort in this cohort caused by microvascular disease.

The main coronary wave energy responsible for coronary blood flow deceleration and flow impediment during systole is the backward compression wave. In FL coronary disease, the BCW strongly correlated with LV contraction as measured by dP/dT max during rest in all patients. Preceding experimental data demonstrate that dP/dt max is the most sensitive measure of contractility but also the most variable with regard to loading conditions (Asrress et al., (2017)). Due to our small patient cohort, although a significant difference in ESPVR/or Ees was not observed, we did identify an increase in dP/dt max in the control group on exercise, likely due to better sensitivity. This would suggest that the increasing force of systolic contraction directly impacts coronary blood flow velocity transmitted as the BCW energy.

### 4.1 Limitations

This was a single center study, and due to the associated technical challenges of simultaneous pressure–volume and coronary pressure and flow measurements, the number of participants was small. Global rather than regional LV function was measured due to the impact of noise created by exercise on PV loop measurements; however, pre‐clinical studies have demonstrated that the impact of single vessel coronary disease can be measured by the effects on the global LV function (Steendijk et al., 1998). To accurately measure cardiac efficiency, the measurement of myocardial oxygen consumption by coronary sinus sampling would be required, which was not performed; therefore, measures of cardiac efficiency are estimations based on pressure–volume loop measurements. Larger numbers would be required to confirm a true correlation between BCW energy and dP/dTmax. Estimates of Ees (and therefore the ration of Ea:Ees) are limited by assuming V0 to be 0 and using single point calculations; however, we were not able to perform IVC occlusion in awake human participants. Furthermore, equations based on single‐beat calculations have not been validated in exercise, and whilst we attempted to perform these, they produced erroneous non‐physiological results. VA coupling is presented and interpreted as the ratio of Ea to Ees consistent with previous clinical reports, and this can alternatively be presented as Ees:Ea, which may better represent the adaptation of contractility to afterload.

## 5 CONCLUSIONS

This study demonstrates that in the presence of FL CAD, exercise can lead to impairment of LV contractile function and a deterioration in VA coupling compared to a control cohort. Further work with direct measures of myocardial oxygen consumption may further our knowledge of the impact of CAD on cardiac efficiency. A greater understanding of the mechanisms of ischemia facilitates development of targeted therapies in this cohort.

## 6 CONFLICT OF INTEREST STATEMENT

The authors have no disclosures or conflicts of interest to share.

## AUTHOR CONTRIBUTION

Substantial contributions to the conception or design of the work: TP, MSM, SR, JL, DP, and SRiv. Acquisition, analysis, or interpretation of data for the work: TP, SA, RW, NB, KA, CA, DB, JS, AP, BC, JJ, HE, and HZRM. Drafting the work or revising it critically for important intellectual content: TP, DB, JS, SR, MSM, JL, and CA.
